# Supplementary material for: The causal relationship between the human gut microbiota and pyogenic arthritis: a Mendelian randomization study
Source: Front Cell Infect Microbiol. 2024 Nov 26;14:1452480. doi: 10.3389/fcimb.2024.1452480 (PMC11629706; doi:10.3389/fcimb.2024.1452480)
Supplement: Supplementary file 1 [file DataSheet1.zip › mendelian test/ebi-a-GCST90027509.csv_leave_one_out.pdf]

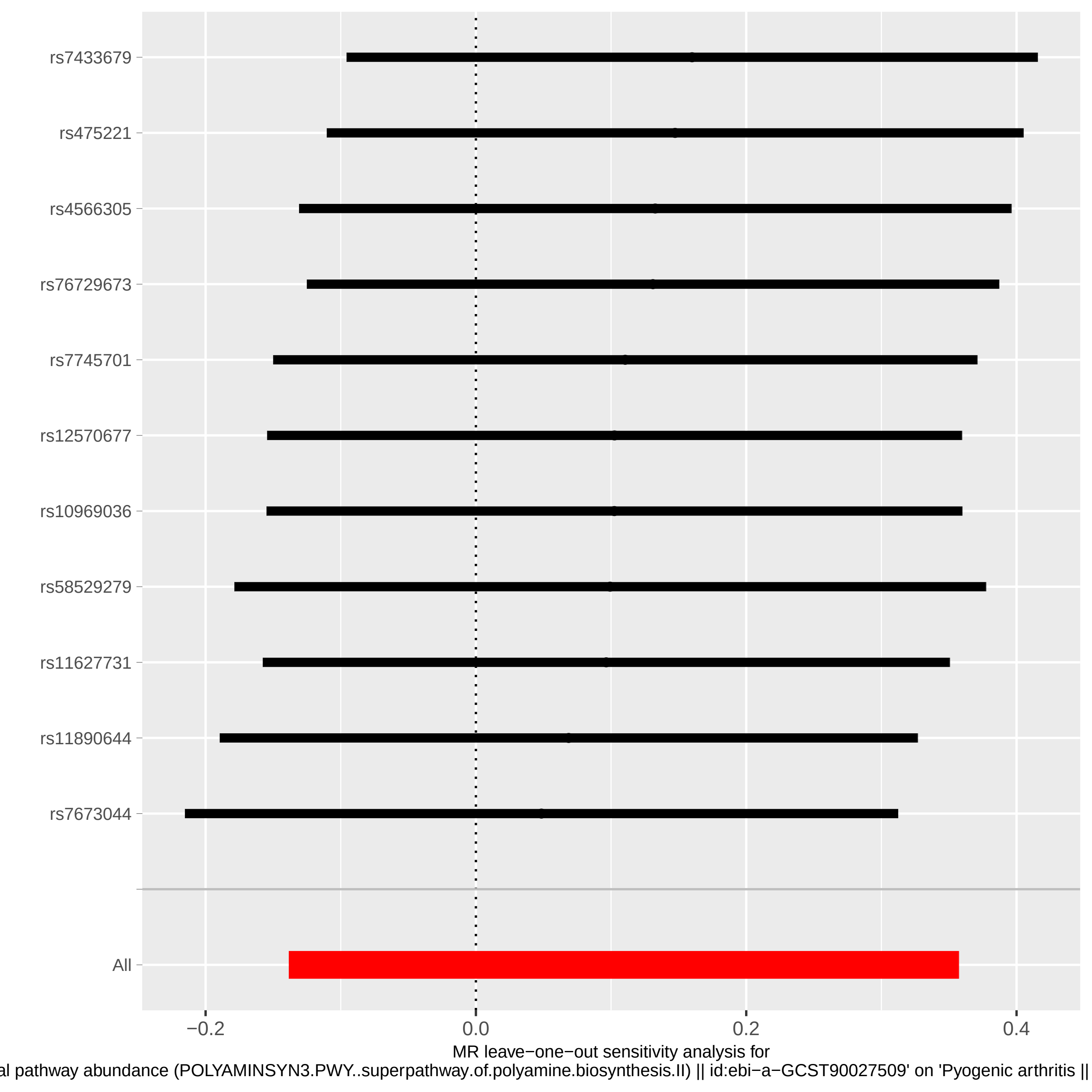
rs7433679
rs475221
rs4566305
rs76729673
rs7745701
rs12570677
rs10969036
rs58529279
rs11627731
rs11890644
rs7673044
All
−0.2
0.0
0.2
0.4
MR leave−one−out sensitivity analysis for
al pathway abundance (POLYAMINSYN3.PWY..superpathway.of.polyamine.biosynthesis.II) || id:ebi−a−GCST90027509' on 'Pyogenic arthritis ||
